# The *BRCA1* exon 13 duplication: clinical characteristics of 22 families in Northern Sweden

**DOI:** 10.1007/s10689-018-0098-y

**Published:** 2018-08-22

**Authors:** Christina Edwinsdotter Ardnor, Anna Rosén, Ingrid Ljuslinder, Beatrice Melin

**Affiliations:** Department of Radiation Sciences, Oncology, Umeå University, Umeå, Sweden

**Keywords:** *BRCA1*, *BRCA1* ins6kbEx13, Breast cancer, Ovarian cancer, Prostate cancer, Colon cancer

## Abstract

The clinical management of *BRCA1*/*2* mutation carriers requires accurate cancer risk estimates. Cancer risks vary according to type and location of the mutation and since there is limited information about mutation-specific cancer risks, genotype-phenotype correlation studies are needed. This is a report of 22 families with the same mutation, *BRCA1* duplication exon 13, a mutation that is found world-wide, with the objective to describe the cancer history found in these families. We studied 69 confirmed carriers, 53 women and 16 men, and additionally 29 women who were clinically expected carriers. Among the confirmed carriers, 27 women (51%) were diagnosed with breast cancer, 10 (19%) with ovarian cancer, 5 (9%) with breast and ovarian cancer and 17 (32%) without cancer. Nine women (17%) with breast cancer were 35 years or younger at diagnose. Also, two cases of early onset colon cancer were found, and 37,5% of the male carriers were diagnosed with prostate cancer. These data may have implications for risk assessment and cancer prevention decision making for carriers of the *BRCA1* duplication exon 13 mutation.

## Introduction

A germline pathological variant in the *BRCA1* or *BRCA2* genes confers to a high lifetime risk of both breast and ovarian cancer, and when such a variant is confirmed, adequate strategies for surveillance can be recommended. Surveillance includes screening with mammography and/or MRI of the breast, and risk-reducing surgery—mastectomy and/or salpingo-oophorectomy.

Counselling families about their cancer risk can be challenging due to wide-ranging risk estimates. In 1995, Easton et al. [1] reported an estimated breast cancer risk for *BRCA1* carriers of > 80% while more recent data estimate an average cumulative risk by age 70 years to be 60% for breast cancer, 59% for ovarian cancer and 83% for contralateral breast cancer [2]. However, it has also been shown that the cancer risk varies by type and location of *BRCA1* mutation [3].

In Northern Sweden, we note a high proportion of a specific mutation in *BRCA1*, duplication exon 13. In this report, we describe the phenotype of this specific *BRCA1* variant. This mutation is a large genomic rearrangement, the 4:th most common *BRCA1* mutation in North America [4] and comprise for 9% of *BRCA1* mutations in UK [5]. It has also been found in other geographically diverse populations, such as Australia, Belgium, Canada, Italy, Norway [5, 6]. We herein describe clinical data on 98 individuals from 22 different families.

## Materials and methods

At the cancer genetic clinic at the University Hospital of Umeå, we identified 22 families during 1998–2017, with at least one confirmed carrier of the *BRCA1*dup ex13, also known as *BRCA1* ins6kbEx13 or according to HGVS: C.4186 − 1787_4357 + 4122dup. These 22 families comprise 15% of all *BRCA1* mutations registered at our clinic.

Within the 22 families there was 91 confirmed mutation screened carriers or obligate carriers. To give a comprehensive picture of the clinical presentation of the families we also included 29 clinically expected carriers from previous generations where no blood sample was available to ascertain mutation status, rendering a total of 120 individuals.

The definition of a clinically expected carrier was women with a verified diagnose of breast or ovarian cancer where at least one family member was a confirmed carrier of the *BRCA1*dupex13.

In the total group of 120 individuals, 71 were deceased, and these individuals were included without contacting relatives for informed consent. To obtain further clinical information about the breast and ovarian cancer cases from the regional quality registry and to verify additional cancer diagnoses by the Swedish Cancer Registry we proceeded to contact the 49 living individuals. 11 were excluded when their addresses could not be ascertained. The 38 remaining individuals were contacted for written informed consent, and the 11 who failed to answer were excluded. After the exclusion, the confirmed carrier group consisted of 69 individuals, and in the total group also including clinically expected carriers, 98 individuals remained.

For families investigated from 2008 to 2017 the BOADICEA calculated mutation probability prior to mutation screening was obtained. For families investigated prior to 2008, we supplemented with calculation of mutation probability according to the information known in the family at time of mutation screening, using BOADICEA WEB APPLICATION version 3.

The Regional Ethical Review Board in Umeå approved the study.

## Results

To give a comprehensive picture of the clinical presentation of the families, we chose to present the number of cancer diagnoses per family, including age at diagnose, from the total group of all 120 individuals, both verified carriers, obligate carriers and clinically expected carriers, in Table 1. We then present more detailed data obtained after informed consent in Table 2.

**Table 1:** Cancer incidence in families with *BRCA1*ins6kbEx13 mutation

Family	No of individuals, confirmed and expected carriers N = 120*	Breast cancer, no of diagnoses, age at onset (year)	Ovarian cancer, no of diagnoses, age at onset (year)	Other cancer types, age at onset (year)	BOADICEA brca1/brca2** (%)
1	2	**1** 50	0	Prostate, 62	3.3
2	4	**3** 44, 50, 58	0	Parathyroid, 38	6.4
3	2	**2** 44, 52	0	Prostate, 73	6.8
4	1	**2** 31, 47	0	0	7.0
5	4	**1** 53	**1** 48	Squamous cell cancer, 84	10.2
6	7	**2** 31, 42	0	Colon 47	20.1
7	5	**3** 35, 46, 55	**1** 68	0	34.8
8	5	**4** 37, 40, 42, 43	**1** 48	0	46.7
9	3	**5** 31, 43, 51, 59, 70	0	0	49.2
10	6	**2** 28, 46	**1** 48	Prostate, 58 Lung/bronch, 69 Prostate, 74	60.7
11	3	**3** 43, 48, 63	**2** 63, 70	0	60.8
12	10	**2** 31, 47	**1** 38	Colon, 42 Papilla Vateri, 52	63.0
13	9	**4** 38, 39, 45, 53	**2** 51, 55	Lung/bronch, 63	65.4
14	2	**2** 28, 53	**1** 58	0	71.0
15	9	**6** 31, 42, 42, 43, 45, 48	0	0	71.4
16	4	0	**4** 49, 67, 75, 78	0	73.4
17	3	**4** 24, 34, 59, 69	0	Lung/bronch, 56	79.7
18	5	**2** 32, 35	**2** 50, 55	Pancreas, 69	87.3
19	19	**7** 34, 42, 50, 51, 58, 61, 65	**3** 44, 62, 62	Cervix, 25 Cervix, 41	89.4
20	5	**6** 43, 47, 49, 51, 53, 55	**1** 50	0	91.0
21	9	**1** 48	**1** 41	Prostate, 57 Prostate, 69	91.2
22	3	**1** 56	**3** 40, 46, 58	0	98.5

Bold numbers indicate number of diagnoses of breast cancer or ovarian cancer in the families respectively

Number of confirmed cancer diagnoses per family, including age at onset, and BOADICEA estimation of mutation probability prior to mutation screening

*All individuals before exclusion

**Estimation of mutation probability prior to screening calculated with BOADICEA WEB APPLICATION version 3. Sensitivity 0.9 for both BRCA1 and BRCA2 and using Swedish cancer incidence rates. Results for BRCA1 and BRCA2 combined

**Table 2:** Characteristics of BRCAins6kbEx13-carriers (n = 69) and pooled data of BRCAins6kbEx13-carriers and clinically expected carriers (expected carriers n = 29, in total n = 98)

	BRCA1ins6kbEx13 carrier No. = 69	Confirmed carriers including clinically expected carriers No. = 98
Women	53	82
Men	16	16
Breast cancer		
No of women with BC diagnose, No. (%)	27 (51)	43 (52)
No of men with BC diagnose, No	0	0
No of Breast cancer diagnoses, No	33	53
Bilateral BC, No. (%)	3 (6)	7 (9)
Two BC diagnoses, same side, No. (%)	3 (6)	3 (4)
Mean age at first BC diagnose, year (range)	42,4 (24–65)	45,0 (24–69)
TNBC, *No. (%)	10 (30)	11 (21)
ER pos, Her2 neg BC, No	1	2
ER pos, Her2 pos BC, No	4	4
ER neg, Her2 pos BC, No	2	2
BC, ER or Her2 receptor status not known, No. (%)	16 (48)	34 (64)
Ovarian cancer		
No of women with ovarian cancer diagnose, No (%)	10 (19)	24 (29)
Mean age at ovarian cancer diagnose, year (range)	57.4 (41–70)	55.2 (38–78)
Ovary, serous, No	5	9
Ovary, endometrioid, No	1	1
Ovary, adenocarcinoma, NOS, No	2	2
Ovary, type not known, No	2	12
Both Breast and Ovarian cancer		
No of individuals with both diagnoses (%)	5 (9)	7 (9)
Other cancer diagnoses among confirmed carriers		
No of other cancer diagnoses (female/male)	14 (5/9)	
Female, cervix uteri	2	
Female, colon (age, year)	2 (42. 47)	
Male, prostate (range, year)	6 (57–74)	
Male, lung/bronch	2	
Male, Papilla Vateri	1	

*TNBC Triple Negative Breast Cancer

Using BOADICEA to calculate Mutation probabilities before mutation screening revealed a broad range estimate from 3.3 to 98.5% (Table 1).

Cancer characteristics for both groups are presented in Table 2. Among confirmed carriers, 27 (51%) of the women were diagnosed with breast cancer, and nine of them (17%) were 35 or younger at first breast cancer diagnose, with two cases in very early age, 24 and 28 years. When excluding five individuals with both breast and ovarian cancer, 8 out of 22 (36%) died due to breast cancer (bc), the mean time between bc diagnose and death was 3.9 years. In many cases, especially in the group including clinically expected carriers, information about Estrogen receptor status or Her2 receptor status was missing, but all histopathological types of breast cancer were present. All individuals who were diagnosed with both ovarian and breast cancer had their breast cancer diagnosed two years or more before their ovarian cancer.

**Fig. 1:**
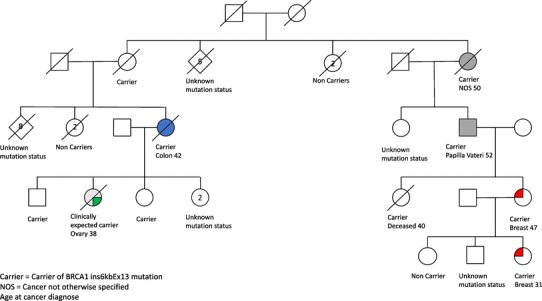
Family with colon cancer, illustrated after exclusion

**Fig. 2:**
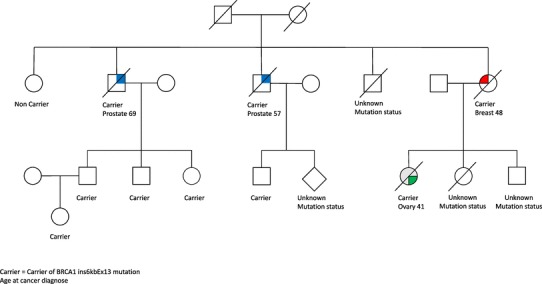
Family with prostate cancer, illustrated after exclusion

Of the women in the confirmed carrier group (n = 53), 17 (32%) had no cancer diagnose. 18 (34%) died of cancer; eight of breast cancer, eight of ovarian cancer and two from colon cancer. The two female carriers with colon cancer came from two different families and were diagnosed at age 42 and 47, and both died within one year of diagnose. Among male carriers (n = 16), six (37,5%) were diagnosed with prostate cancer and there were also two cases of lung cancer at age 63 and 69, and one case of cancer arising from Papilla Vateri at age 52 (Table 2). Two families are illustrated with pedigrees, one including colon cancer (Fig. 1), and one with prostate cancer (Fig. 2).

Since clinical data was known in the families, we could assess that the exclusion of the individuals who we could not reach to ask for informed consent, and the ones who failed to answer, (in total 22 individuals), did not markedly influence the results.

## Discussion

This is, to our knowledge, the largest cohort of *BRCA1* duplication exon 13 mutation carriers. 19 of the 22 families originate from Northern Sweden, but from different geographical areas in this region. Our findings are consistent with the data from James et al. that showed that large genomic rearrangements in *BRCA1* are associated with an increased risk of high risk features, such as bilateral breast cancer, diagnosis < 40 years and ovarian cancer [7]. Our results show a high proportion of early onset breast cancer, and since the best cost-benefit of prophylactic mastectomy is shown when surgery is performed before first breast cancer diagnose this might be worth considering when counselling women with this mutation.

The elevated risk for prostate cancer in *BRCA2* carriers is well known. However, a recent study of 1072 *BRCA1*/*2* mutation carriers by Mersch et al. did not show an increased prostate cancer risk for *BRCA1* mutation carriers [8] and there is to date no recommendation for prostate cancer screening in this group. Interestingly, we found that 37,5% of the male carriers in our cohort were diagnosed with prostate cancer in comparison to the expected 17% in the Swedish population [9]. This might suggest that in *BRCA1* ins6kbEx13 families’ male carriers should be recommended PSA screening according to practice in families with hereditary elevated risk.

When counselling families, the mutation probability tool BOADICEA has become a valuable asset. Our families show a wide range of mutation probability prior to screening. Therefore, BOADICEA should be seen as a tool for support but if there is limited information due to a small pedigree or loss of relevant information, mutation screening in a breast-ovarian cancer family should be recommended in spite of low BOADICEA estimates.

We found two women with early onset colon cancer, both with fatal outcome. When reviewing colorectal cancer risk in 7015 female *BRCA1* and *BRCA2* mutation carriers, Sopik et al. found a significant fivefold increased risk of colorectal cancer for female *BRCA1* mutation carriers under age 50 (Sopik et al. [10]). Since colorectal cancer can be prevented through colonoscopy screening and removal of adenomatous polyps the authors proposed that all female *BRCA1* mutation carriers should be offered colonoscopy at 3- to 5-year intervals between the ages of 40 and 50 years. As colon cancer is rarely occurring in the families this might be a too strong recommendation, but our data suggest that it may be relevant for in example the *BRCA1* ins6kbEx13 mutation. In conclusion, we here present a large case series of *BRCA1* dup ex13 mutation carriers that present with early onset breast and ovarian cancer, but we also see a higher than expected rate of prostate and early onset colorectal cancer implying that additional surveillance may be necessary for carriers of this mutation.
